# Exposure to Metal-Rich Particulate Matter Modifies the Expression of Candidate MicroRNAs in Peripheral Blood Leukocytes

**DOI:** 10.1289/ehp.0901300

**Published:** 2010-01-08

**Authors:** Valentina Bollati, Barbara Marinelli, Pietro Apostoli, Matteo Bonzini, Francesco Nordio, Mirjam Hoxha, Valeria Pegoraro, Valeria Motta, Letizia Tarantini, Laura Cantone, Joel Schwartz, Pier Alberto Bertazzi, Andrea Baccarelli

**Affiliations:** 1 Center of Molecular and Genetic Epidemiology, Department of Environmental and Occupational Health, Università degli Studi di Milano and IRCCS Fondazione Ca’ Granda Ospedale Maggiore Policlinico, Milan, Italy; 2 Department of Experimental and Applied Medicine, Occupational Medicine and Industrial Hygiene, University of Brescia, Brescia, Italy; 3 Department of Clinical and Biological Sciences, University of Insubria, Varese, Italy; 4 Department of Clinical Medicine, Nephrology and Health Sciences, University of Parma Medical School, Parma, Italy; 5 Exposure, Epidemiology and Risk Program, Department of Environmental Health, Harvard School of Public Health, Boston, Massachusetts, USA

**Keywords:** epigenetics, etiology, miRNA expression, particulate matter, peripheral blood leukocytes

## Abstract

**Background:**

Altered patterns of gene expression mediate the effects of particulate matter (PM) on human health, but mechanisms through which PM modifies gene expression are largely undetermined. MicroRNAs (miRNAs) are highly conserved, noncoding small RNAs that regulate the expression of broad gene networks at the posttranscriptional level.

**Objectives:**

We evaluated the effects of exposure to PM and PM metal components on candidate miRNAs (*miR-222, miR-21*, and *miR-146a*) related with oxidative stress and inflammatory processes in 63 workers at an electric-furnace steel plant.

**Methods:**

We measured *miR-222*, *miR-21*, and *miR-146a* expression in blood leukocyte RNA on the first day of a workweek (baseline) and after 3 days of work (postexposure). Relative expression of miRNAs was measured by real-time polymerase chain reaction. We measured blood oxidative stress (8-hydroxyguanine) and estimated individual exposures to PM_1_ (< 1 μm in aerodynamic diameter), PM_10_ (< 10 μm in aerodynamic diameter), coarse PM (PM_10_ minus PM_1_), and PM metal components (chromium, lead, cadmium, arsenic, nickel, manganese) between the baseline and postexposure measurements.

**Results:**

Expression of *miR-222* and *miR-21* (using the 2^−ΔΔC_T_^ method) was significantly increased in postexposure samples (miR-222: baseline = 0.68 ± 3.41, postexposure = 2.16 ± 2.25, *p* = 0.002; miR-21: baseline = 4.10 ± 3.04, postexposure = 4.66 ± 2.63, *p* = 0.05). In postexposure samples, *miR-222* expression was positively correlated with lead exposure (β = 0.41, *p* = 0.02), whereas *miR-21* expression was associated with blood 8*-*hydroxyguanine (β = 0.11, *p* = 0.03) but not with individual PM size fractions or metal components. Postexposure expression of *miR-146a* was not significantly different from baseline (baseline = 0.61 ± 2.42, postexposure = 1.90 ± 3.94, *p* = 0.19) but was negatively correlated with exposure to lead (β = −0.51, *p* = 0.011) and cadmium (β = −0.42, *p* = 0.04).

**Conclusions:**

Changes in miRNA expression may represent a novel mechanism mediating responses to PM and its metal components.

Exposure to ambient particulate matter (PM) has been associated with increased morbidity and mortality from cardiovascular and respiratory diseases (Baccarelli et al. 2008; Brook et al. 2004; Ciocco and Thompson 1961). Epidemiologic and *in vivo* studies suggest that the transition metal components of PM may be responsible for such effects (Brook et al. 2004; Chang et al. 2005; Corey et al. 2006). Foundry work has also been associated with adverse health outcomes, including cardiovascular disease (Kuo et al. 1999), potentially linked with PM exposure. Although prior studies have associated inhalation of ambient or occupational PM with systemic activation of inflammatory pathways, production of reactive oxygen species (ROS), and enhanced coagulation (Baccarelli et al. 2007, 2008; Chahine et al. 2007; Gurgueira et al. 2002; Li et al. 2006), the underlying mechanisms linking PM exposure with adverse health outcomes still need to be clarified (Nel et al. 2006).

Inhaled PM pollutants have been shown to produce systemic changes in gene expression, which can be detected in peripheral blood of exposed individuals (Wang et al. 2005). Expression of human genes is controlled by several genetic and epigenetic mechanisms, including microRNA (miRNA) regulation (He and Hannon 2004). MiRNAs are small, endogenous, single-stranded noncoding RNAs of 20–22 nucleotides (Baccarelli and Bollati 2009). They posttranscriptionally regulate gene expression by hybridization to messenger RNA (mRNA), leading to translational repression or degradation of the target mRNA (He and Hannon 2004). One single miRNA can regulate hundreds of mRNAs in interrelated gene pathways, and a single mRNA can be targeted by several different miRNAs (Lewis et al. 2005).

Changes in the expression of several miRNAs, including *miR-222*, *miR-21*, and *miR-146a*, have been implicated in disease mechanisms that may be related to PM exposure, such as oxidative stress (Babar et al. 2008) and regulation of inflammation (Xiao and Rajewsky 2009). In particular, *miR-222* overexpression indirectly reduces the expression of the endothelial nitric oxide synthasein Dicer small interfering RNA–transfected cells (Suarez et al. 2007), an inflammation-related hallmark of atherosclerosis and ischemic cardiomyopathy (Zeiher 1996), and has been associated with altered redox signaling (Sen et al. 2009). *miR-21* has been shown to respond to hydrogen peroxide stimulation and participates in coordinated protective responses to oxidative stress (Cheng et al. 2009), as well as in inflammatory responses as suggested by animal models of allergic airway (Lu et al. 2009) and lipolysaccharide-induced inflammation (Moschos et al. 2007). Changes in *miR-146a* expression have been implicated in the negative regulation of inflammation induced via the innate immune response, which is also activated by PM (Shoenfelt et al. 2009). *miR-146a* expression during inflammation is under the control of the transcription factor NF-κB (nuclear factor-kappa B), a central mediator for PM-related inflammation and health effects (Williams et al. 2008).

In the present study, we investigated the effects of PM exposure on miR-222, miR-21, and miR-146a measured in blood RNA from foundry workers with well-characterized exposure to a wide range of PM levels. To clarify the mechanisms activated by PM exposure, we evaluated whether the expression of *miR-222*, *miR-21*, and *miR-146a* was associated with oxidative stress levels, as reflected in 8-hydroxyguanine (8-OH-dG) measured in blood DNA.

## Materials and Methods

### Study subjects

We recruited 63 healthy male workers (mean age, 44 years; range, 27–55 years), in a steel production plant in Brescia, Northern Italy. These workers were free of cancer, cardiovascular disease, and pulmonary disease, and all of them had been working in their current job position for at least 1 year. To discriminate short- and long-term effects of PM, we obtained blood samples for DNA methylation analysis at two different times: *a*) a baseline sample collected in the morning of the first day of a workweek (after 2 days off work) before the beginning of any work activity (time 1); and *b*) a postexposure sample collected at the same time on the fourth day of work, after 3 consecutive days of work (time 2). Written informed consent and approval from the local institutional review board IRCCS Fondazione Ca’ Granda Ospedale Maggiore Policlinico were obtained before the study.

### Exposure assessment

Measures of airborne PM mass and PM metal components obtained in each of the 11 work areas of the steel production facility were used to estimate individual exposures. Measures of PM mass included levels of PM with aerodynamic diameters ≤ 10 μm (PM_10_) and ≤ 1 μm (PM_1_) measured using a GRIMM 1100 light-scattering dust analyzer (Grimm Technologies, Inc., Douglasville, GA, USA). Concentrations of coarse particles were calculated from these measures as the difference between PM_10_ and PM_1_. We measured PM metal components on the PM_10_ fraction of PM mass through multielemental analysis by means of inductively coupled plasma mass spectrometer (ELAN DRC II; PerkinElmer, Waltham, MA, USA). We measured arsenic, cadmium, lead, manganese, and nickel concentrations using the Total Quant method, and chromium concentrations using the Dynamic Reaction Cell (DRC) method with ammonia (De Palma et al. 2008).

During the 3 working days between times 1 and 2, each of the study subjects recorded in a personal log the time that he spent in each work area. Individual exposures were calculated as the time-weighted averages of area concentrations (Tarantini et al. 2009).

### miRNA analysis

Buffy coat samples were separated within 30 min of blood draw, immediately snap-frozen, and stored at −80°C. RNA was extracted from the buffy coats using the Ribopure Kit (Ambion, Inc., Austin, TX, USA), modified for miRNA extraction [see Supplemental Material (doi:10.1289/ehp.0901300)]. We used specific TaqMan MicroRNA Assays (Applied Biosystems, Uppsala, Sweden) to detect and quantify mature miRNAs as recommended by the manufacturer, using ABI Prism 7900HT sequence detection systems (Applied Biosystems). In the reverse transcription (RT) step, 10 ng total RNA was employed in RT reactions (16°C for 30 min, 42°C for 30 min, 85°C for 5 min, and then to 4°C) using reagents from the TaqMan MicroRNA Reverse Transcription kit (Applied Biosystems) and specific miRNA primers provided with the TaqMan MicroRNA Assays. Real-time polymerase chain reaction (PCR) was performed using TaqMan MicroRNA Assays together with TaqMan Universal PCR Master Mix on an Applied Biosystems 7900 Sequence Detection System (95°C for 1 min and 40 cycles of 95°C for 15 sec and 60°C for 30 sec) (Chen 2005). Normalization was performed with *RNU6B* (RNA, U6 small nuclear 2) endogenous control (Applied Biosystems, Foster City, CA, USA). Real-time PCR was performed in triplicate, including no-template controls. The threshold cycle (Ct) was defined as the fractional cycle number at which the fluorescence passes the fixed threshold. The relative gene expression was calculated via a 2^−ΔΔC_t_^ method (Livak and Schmittgen 2001). Data are presented as relative quantity of target miRNA, normalized to *RNU6B* endogenous control and a calibrator built as a pool of 50 random samples. All laboratory analyses were performed in the same batch using samples that remained frozen until the analyses.

### Determination of 8-OH-dG content in mitochondrial DNA (mtDNA)

We measured the content of 8-OH-dG in mtDNA using quantitative real-time PCR to amplify different fragments of mtDNA on each sample with and without hOGG1 (human 8-oxoguanine DNA glycosylase 1) pretreatment, as previously described by Lin et al. (2008). All experimental DNA samples were assayed in triplicates, and the mean value of ΔC_t_ was used in the statistical analyses.

### Target prediction and pathway mining

We used the miRNA target prediction software miRanda (http://www.microrna.org/microrna/home.do) to predict the target genes of *miR-222*, *miR-21*, and *miR-146a*. A total of 1,056 target genes were annotated for *miR-222*, 1,065 for *miR-21*, and 1,038 for *miR-146a*. Kyoto Encyclopedia of Genes and Genomes (KEGG; http://www.genome.jp/kegg) pathway searching was performed by mapping the predicted target genes from the KEGG human database. A total of 150 KEGG pathways were annotated for *miR-222*, 153 for *miR-21*, and 160 for *miR-146a*.

To find signal transduction pathways related to *miR-222*, *miR-21*, and *miR-146a*, we used LitInspector (http://www.litinspector.org), a literature search tool providing gene and signal transduction pathway mining within the National Center for Biotechnology Information’s PubMed database (Frisch et al. 2009). Two LitInspector pathways were annotated for *miR-222*, seven for *miR-21*, and six for *miR-146a*.

### Statistical analysis

The Student’s paired *t-*test was used to assess differences in miRNA expression between baseline (time 1) and postexposure (time 2). We evaluated the association of PM mass and PM metal component levels with miRNA expression measured in postexposure samples using simple linear regression models and multivariable models. The adjusting variables were selected *a priori* based on the assumption that potential confounders had to be related to inflammation or oxidative stress generation. Age, body mass index (BMI), smoking, and number of cigarettes per day are all potential determinants of inflammation, oxidative stress, or both. In addition, we adjusted for percent granulocytes to control for possible shifts in leukocyte differential count.

As a sensitivity analysis, we also fitted duration of smoking in the model described above. The results of the models including duration of smoking were very similar to those including number of cigarettes per day.

To compare the magnitude of the associations of miRNA expression with different exposures, we calculated standardized regression coefficients. Outliers were excluded from regression analysis by dropping observations with studentized residuals that exceeded +3 or −3. We checked regression assumptions by performing diagnostic tests for each model, including the Shapiro–Wilk test to verify normality of residuals and the White test to verify the homogeneity of variance of the residuals. For miRNAs that did not show differences in their expression between time 1 and time 2, we also used mixed models that regressed PM and metal exposures against all measures of miRNA expression, regardless of whether they were measured at baseline or postexposure samples (Tarantini et al. 2009). A two-sided *p*-value < 0.05 was considered statistically significant. All statistical analyses were performed using SAS (version 9.1.3; SAS Institute Inc., Cary, NC, USA).

## Results

### Subject characteristics and exposure

The characteristics of the 63 study subjects are summarized in Table 1. miRNA expression did not show significant correlations with any of these characteristics [see Supplemental Material, Table S1 (doi:10.1289/ehp.0901300)]. Supplemental Material, Table S2, shows the average levels and distributions of the individual exposures to PM and PM metal components during the 3 workdays between the baseline and postexposure miRNA measurements.

### Differences in miR-222, miR-21, and miR-146a expression between baseline (time 1) and postexposure (time 2)

**Figure 1:**
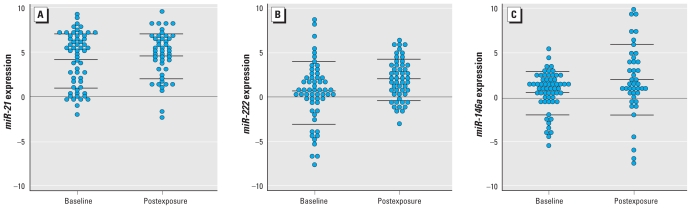
Dot plots representing baseline and postexposure measures of *miR-21* (*A*), *miR-222* (*B*), and *miR-146a* (*C*) expression. Data are represented as −ΔΔ^C_t_^ to approximate the Gaussian distribution; for postexposure compared with baseline, *p* = 0.0503 for *miR-21* (*A*), *p* = 0.0024 for *miR-222* (*B*), and *p* = 0.1917 for *miR-146a* (*C*).

*miR-222* expression was significantly increased in blood samples taken after 3 days of work (time 2) compared with the measurement at time 1 [mean ± SD: mean_time1_ = 0.68 ± 3.41; mean_time2_ = 2.16 ± 2.25; *p* = 0.002; Figure 1]. *miR-21* expression was also significantly increased in postexposure samples (mean_time1_ = 4.1 ± 3.04; mean_time2_ = 4.7 ± 2.63; *p* = 0.05). *miR-146a* was not significantly different between baseline and postexposure blood samples (mean_time1_ = 0.61 ± 2.42; mean_time2_ = 1.9 ± 3.94; *p* = 0.19).

### Associations of individual exposure levels to PM and PM metal components with miR-222, miR-21, and miR-146a expression

*miR-222* expression exhibited a positive association with the levels of lead exposure [unadjusted analysis: β_std_ = 0.33; 95% confidence interval (CI), 0.07 to 0.76; *p* = 0.02; multivariable regression: β_std_ = 0.41; 95% CI, −0.01 to 0.67; *p* = 0.06, adjusting for age, BMI, current smoking, number of cigarettes per day, and percent granulocytes].

*miR-21* expression was not significantly associated with any of the measures of exposure to PM and PM metal components (Table 2).

*miR-146a* expression showed a negative significant association with lead exposure (unadjusted models: β_std_ = −0.51; 95% CI, −0.88 to −0.14; *p* = 0.008; multivariable regression: β_std_ = −0.51; 95% CI, −0.88 to −0.13; *p* = 0.011; Table 2). *miR-146a* expression also showed a negative significant association with cadmium (unadjusted models: β_std_ = −0.41; 95% CI, −0.74 to −0.07; *p* = 0.019; multivariable regression: β_std_ = −0.42; 95% CI, −0.83 to −0.02; *p* = 0.043; Table 2). Because, as shown in Figure 1, the mean *miR-146a* expression in the baseline and postexposure blood samples did not show significant differences, we also evaluated the associations with lead and cadmium exposures in mixed regression models that included both sets of *miR-146a* expression measurements as the dependent variable (pooled data analysis of baseline and postexposure *miR-146a* expression). In these models, we evaluated the association between the exposure and *miR-146a,* assuming that there were no short-term effects from the exposures (i.e., the association between the exposure and *miR-146a* was similar at both time points, potentially reflecting persistent effects of the usual, long-term condition of exposure). In these models, *miR-146a* expression showed a negative significant association with lead exposure (unadjusted models: β = −0.15; 95% CI, −0.26 to −0.03; *p* = 0.01; multivariable regression: β = −0.15; 95% CI, −0.27 to −0.03; *p* = 0.01). *miR-146a* expression also showed a negative significant association with cadmium (unadjusted models: β = −115.80; 95% CI, −236.33 to 4.73; *p* = 0.06; multivariable regression: β = −142.51; 95% CI, −284.00 to −1.03; *p* = 0.05).

### Associations of miR-222, miR-21, and miR-146a expression with 8-OH-dG

In univariate analysis, we found a positive correlation between *miR-21* expression and 8-OH-dG (β = 0.11; 95% CI, 0.01–0.21; *p* = 0.03), which was confirmed in multivariable regression (β = 0.11; 95% CI, 0.02–0.20; *p* = 0.01). *miR-222* and *miR-146a* were not associated with 8-OH-dG (Table 2).

### Associations between exposures and miRNA expression stratified by smoking and age

In noncurrent smokers, *miR-222* expression was up-regulated in response to chromium (β = 37.09; 95% CI, 13.72 to 60.46; *p* = 0.003), lead (β = 0.19; 95% CI, 0.03 to 0.34; *p* = 0.020), and cadmium (β = 141.34; 95% CI, 7.32 to 275.36; *p* = 0.039), whereas in current smokers we observed no significant association. In noncurrent smokers, *miR-146a* expression was down-regulated in response to lead (β = −0.58; 95% CI, −0.87 to −0.29; *p* < 0.001) and cadmium (β = −414.26; 95% CI, −726.87 to −101.64; *p* = 0.01), whereas in current smokers we observed no significant association.

The results stratified by age showed that in subjects 25–45 years of age *miR-222* was up*-*regulated in association with lead (β = 0.21; 95% CI, 0.08 to 0.34; *p* = 0.003), whereas *miR-146a* expression was down- regulated in association with lead (β = −0.38; 95% CI, −0.59 to −0.17; *p* = 0.001). We found no significant associations among individuals between 46 and 65 years of age. The complete analyses stratified by smoking and age are reported in Supplemental Material, Tables S3 and S4 (doi:10.1289/ehp.0901300).

### Target mapping and functional analysis: potential interactions of miR-222, miR-21, and miR-146a with signal transduction pathways

To explore the functional significance of the miRNAs investigated, we applied KEGG (http://www.genome.jp/kegg), a pathway analysis database, to the target genes identified for *miR-21*, *miR-222*, and *miR-146a* using miRanda. The enriched pathways we identified appeared to be largely overlapping (i.e., many target genes were in more than one pathway). Among the top-ranked pathways, we found those related to general functions (e.g., purine metabolism, cell cycle) and others with more specific functions, including a high proportion of pathways related to oxidative stress and inflammation [Table 3; for a complete pathway list, see Supplementary Material, Table S5 (doi:10.1289/ehp.0901300)]. Using LitInspector, we scanned PubMed for co-occurrence of the user input gene (*miR-222*, *miR-21*, or *miR-146a*) and the general pathway key words in the same sentence. We found two pathways related to *miR-222*, seven related to *miR-21*, and six related to *miR-146a* (Table 4).

## Discussion

In this study of foundry workers in an electric-furnace steel plant, we evaluated the effect of exposure to PM and PM metal components on the expression of three candidate miRNAs that regulate genes in pathways related to oxidative stress and inflammation. We found that *miR-222* and *miR-21* expression was increased in postexposure samples collected after 3 workdays, compared with baseline samples. *miR-222* expression in postexposure samples was positively associated with the mean lead exposure measured in the PM_10_ fraction of the PM mass during the 3 workdays, whereas we found no significant association of *miR-21* expression with the exposures levels we evaluated. In addition, although *miR-146a* expression did not differ in postexposure and baseline samples, we observed that individual levels of exposure to lead and cadmium in the PM_10_ fraction were associated with *miR-146a* expression in postexposure samples, as well as in a pooled data analysis that included both baseline and postexposure samples.

Although many studies have focused on comparing miRNA expression between pathologic samples and normal tissues, very few studies have evaluated changes in miRNA expression in response to environmental stimuli (Baccarelli and Bollati 2009). A recent *in vitro* study by Jardim et al. (2009) showed that miRNA expression profiles in human airway cells change in response to diesel exhaust particles. In that study, 197 of the 313 detectable miRNAs (62.9%) were either up- or down-regulated ≥ 1.5 times, including many miRNAs associated with responses in inflammatory pathways. To the best of our knowledge, ours is the first human study to show that PM modifies the *in vivo* expression of candidate miRNAs.

*miR-222* expression has been related with nitric oxide (Suarez et al. 2007) and redox signaling (Sen et al. 2009). An up-regulation of *miR-222*, as measured in our study, may suggest an increased proliferation rate of blood leukocytes in response to environmental stimuli that are able to induce inflammation responses.

Expression of *miR-21* is part of a response aimed at limiting injuries from ROS (Cheng et al. 2009). In the present study, we observed an up-regulation of *miR-21* after 3 days of work compared with the baseline measurement. This may reflect a nonspecific response to ROS production in blood due to increased PM-induced oxidative stress (Brauner et al. 2007), as also suggested by the positive association that we observed between *miR-21* expression and 8-OH-dG. However, we found no significant associations between *miR-21* expression and PM or PM metal components. Other unknown biological changes intervening during the 3 workdays—or other unmeasured exposures that are present in foundry facilities, such as heat, carbon monoxide, and nonionizing radiation (Tarantini et al. 2009)—might have modified *miR-21* expression.

Studies in myeloid cells activated by bacterial and fungal components or after exposure to the proinflammatory cytokines tumor necrosis factor α or interleukin-1β have shown that *miR-146a* is involved in limiting inflammatory responses triggered through the innate immune system (Perry et al. 2008; Williams et al. 2008). The negative association of *miR-146a* expression with lead and cadmium levels that we observed in the present study may indicate that PM metal components enhance inflammatory processes initiated by organic PM antigens through the innate system (Descotes 1992; Li et al. 2008).

Bioinformatic strategies, such as those implemented in miRanda software (Bentwich 2005), are now available to identify potential miRNA target sites in the 3′ untranslated region (UTR) of a protein-coding gene. The potential targets of miRNAs often include hundreds of genes because the reverse complement of some “seeds” (bases 2–8 of the mature miRNA) appears in multiple locations in many pre-mRNA 3′ UTRs. With the understanding that recognition of mRNA targets is speculative, we explored *miR-222*, *miR-21*, and *miR-146a* targets and examined whether these targeted genes are overrepresented in pathways annotated in the KEGG database.

miRanda identified > 3,000 genes that are potential targets of the three miRNAs evaluated in our study. Using the KEGG pathway database, we identified several pathways that are involved in immune/inflammatory and oxidative stress. Transition metals are common components of ambient PM and have been shown to interact with the immune system in antigen nonspecific fashion (Mishra 2009). MacNee and Donaldson (2000) proposed that the generation of oxidative stress, either directly by transition metal components of PM or indirectly from the recruitment into the airspaces and activation of blood leukocytes, is a primary mechanism determining the inflammation-related health outcomes of PM. Our results indicate novel pathways through which metals may elicit specific PM-related responses and help explain previous studies that have specifically indicated a role of metals in promoting PM effects (Arisawa et al. 2001; Magari et al. 2002; Messner et al. 2009; Park et al. 2008).

Results of the present study show that associations of metals with *miR-222* and *miR-146a* expression were limited to nonsmokers. In addition, when we divided the study subjects in two subgroups according to age (25–45 years vs. 45–60 years), we observed stronger associations among younger subjects. Identification of sets of individuals who have enhanced responses to PM may suggest possible mechanisms of physiologic assault, and provide data that can be used for more detailed risk assessment (Bateson and Schwartz 2004).

In the present study we investigated a population with well-characterized exposure to PM and PM metal components that allowed for contrasting subjects over a wide range of different exposure levels. Because of the limited number of study subjects, it is possible that the associations observed were due to chance. However, the occupational exposure and relatively controlled environment of a foundry provide a good setting for evaluating these mechanistic questions and reduce bias and chance findings. Our study was based on subjects working in several areas of the same factory but did not include a different population of subjects without exposure to PM. Limiting our investigation to individuals who have all been working in the same facility avoided potential concerns related to the selection of external referents who might have differed from the exposed population in terms of socioeconomic factors and other characteristics determining hiring into the plant (Pearce et al. 2007). However, the differences in the individual levels of exposure within our study group were large, providing sufficient contrast for identifying exposure-related changes in miRNA expression (Anselmi and Patelli 2006).

In summary, our findings suggest that air particles, particularly those rich in lead and cadmium, are able to modify miRNAs expression. Further studies are required to determine the role of such alterations along the pathways determining the effects of PM on human health.

## Figures and Tables

**Table 1:** Characteristics of the study subjects (*n* = 63).

Characteristic	Value
Age (years)	44 ± 7.6
BMI (kg/m^2^)	26.5 ± 2.7
Current smoker	
No	38 (60)
Yes	25 (40)
No. of cigarettes/day	13.0 ± 7.2
Smoking duration (years)	13 ± 12
Duration of employment (years)	16 ± 10
Education	
Primary school (completed grade 5)	12 (19)
Middle school (completed grade 8)	37 (59)
High school (completed)	14 (22)
Area of residence	
City center	8 (13)
Suburb	41 (67)
Rural	12 (20)
Self-reported traffic intensity near home	
High	5 (8)
Medium	38 (62)
Low	18 (30)

Values shown are mean ± SD or *n* (%).

**Table 2:** Unadjusted and multivariable regression models (adjusted for age, BMI, smoking, number of cigarettes/day, percentage of granulocytes) estimating effects of PM mass and PM metal component exposure on *miR-222*, *miR-21*, and *miR-146a* expression (*n* = 63).

	*miR-21*		*miR-222*		*miR-146*	
Exposure	β_std_	95% CI	β_std_	95% CI	β_std_	95% CI
Unadjusted regression						
PM_10_	−0.08	−0.36 to 0.21	0.17	−0.12 to 0.45	−0.58	−1.35 to 0.19
PM_1_	−0.10	−0.41 to 0.21	0.18	−0.18 to 0.45	0.06	−0.40 to 0.52
Coarse PM	−0.07	−0.36 to 0.21	0.17	−0.12 to 0.45	−0.62	−1.40 to 0.16
Chromium	0.02	−0.30 to 0.33	0.29	−0.06 to 0.58	−0.17	−0.48 to 0.15
Lead	−0.08	−0.46 to 0.29	0.33^a^	0.07 to 0.76	−0.51^b^	−0.88 to −0.14
Cadmium	0.31	−0.10 to 0.71	0.10	−0.23 to 0.46	−0.41^c^	−0.74 to −0.07
Arsenic	−0.11	−0.47 to 0.24	−0.03	−0.16 to 0.71	−0.12	−0.49 to 0.25
Nickel	−0.04	−0.44 to 0.36	0.13	−0.18 to 0.61	−0.15	−0.55 to 0.26
Manganese	0.24	−0.06 to 0.53	−0.11	−0.41 to 0.25	−0.03	−0.33 to 0.27
8-OH-dG	0.11^d^	0.01 to 0.21	−0.01	−0.10 to 0.11	0.02	−0.05 to 0.09
Multivariable regression						
PM_10_	−0.03	−0.33 to 0.27	0.16	−0.11 to 0.44	−0.60	−1.48 to 0.27
PM_1_	−0.06	−0.39 to 0.27	0.13	−0.11 to 0.46	0.16	−0.40 to 0.72
Coarse PM	−0.03	−0.33 to 0.27	0.16	−0.11 to 0.44	−0.65	−1.53 to 0.24
Chromium	−0.03	−0.40 to 0.33	0.26^e^	0.00 to 0.58	−0.16	−0.54 to 0.21
Lead	−0.07	−0.49 to 0.35	0.41	−0.01 to 0.67	−0.51^f^	−0.88 to −0.13
Cadmium	0.19	−0.32 to 0.69	0.12	−0.22 to 0.42	−0.42^g^	−0.83 to −0.02
Arsenic	−0.11	−0.57 to 0.36	0.27	−0.38 to 0.32	−0.26	−0.69 to 0.18
Nickel	0.00	−0.45 to 0.45	0.21	−0.25 to 0.50	−0.15	−0.58 to 0.29
Manganese	0.27	−0.09 to 0.63	−0.08	−0.40 to 0.19	−0.04	−0.38 to 0.30
8-OH-dG	0.11^h^	0.02 to 0.20	0.01	−0.11 to 0.08	0.03	−0.03 to 0.10

a *p* = 0.02.

b *p* = 0.008.

c *p* = 0.019.

d *p* = 0.03.

e *p* = 0.05.

f *p* = 0.001.

g *p* = 0.043.

h *p* = 0.01.

**Table 3:** Selected KEGG biological pathways potentially affected by *miR-222*, *miR-21*, and *miR-146a* that have established biological roles in oxidative stress and inflammatory processes.

		No. of target genes involved in the pathway		
Pathway ID	KEGG pathway name	*miR-222*	*miR-21*	*miR-146a*
hsa04010	MAPK signaling pathway	14	11	9
hsa04062	Chemokine signaling pathway	10	9	10
hsa04060	Cytokine-cytokine receptor interaction	7	17	14
hsa04510	Focal adhesion	7	8	10
hsa04210	Apoptosis	6	3	6
hsa04270	Vascular smooth muscle contraction	6	7	6
hsa04660	T cell receptor signaling pathway	6	5	3
hsa04670	Leukocyte transendothelial migration	6	1	5
hsa04620	Toll-like receptor signaling pathway	5	3	7
hsa04662	B cell receptor signaling pathway	5	5	3
hsa04514	Cell adhesion molecules	4	3	1
hsa04350	TGF-beta signaling pathway	3	4	5
hsa04610	Complement and coagulation cascades	3	5	3
hsa00910	Nitrogen metabolism	1	1	1
hsa04370	VEGF signaling pathway	1	2	3
hsa04650	Natural killer cell mediated cytotoxicity	4	8	7

See also Supplemental Material, Table S5 (doi:10.1289/ehp.0901300).

**Table 4:** Signaling pathways potentially related to *miR-222*, *miR-21*, and *miR-146a*, identified by the literature search tool LitInspector.

Pathway component	Signaling pathway	References
*miR-222*		
MAPK	Mitogen activated protein kinase signaling	Terasawa et al. 2009
NGF	Nerve growth factor signaling	Terasawa et al. 2009

*miR-21*		
PTEN	Phosphatase and tension homolog signaling	Meng et al. 2006, 2007; Roy et al. 2009
TGFβ	TGFβ signaling	Davis et al. 2008; Papagiannakopoulos et al. 2008
BMP	TGFβ signaling	Davis et al. 2008
FAK	Focal adhesion kinase 1 signaling	Davis et al. 2008
MAPK	Mitogen activated protein kinase signaling	Thum et al. 2008
P13K	Phosphatidylinositol signaling	Meng et al. 2006
TP53	p53 signaling	Papagiannakopoulos et al. 2008

*miR-146a*		
BCL XL	bcl2 like 1 signaling	Liu et al. 2009
CXCR4	Chemokine c x c motif receptor 4 signaling	Labbaye et al. 2008
EGFR	Epidermal growth factor receptor signaling	Hurst et al. 2009
NFKB	Nuclear factor kappaB signaling	Schmelzer et al. 2009
STAT	Signal transducer and activator of transcription signaling	Liu et al. 2009
TLR	Toll-like receptor signaling	Dai et al. 2008

## References

[b1-ehp-118-763] Anselmi U, Patelli R (2006). Rapporto sulla qualità dell’aria di Brescia e provincia.

[b2-ehp-118-763] Arisawa K, Nakano A, Saito H, Liu XJ, Yokoo M, Soda M (2001). Mortality and cancer incidence among a population previously exposed to environmental cadmium. Int Arch Occup Environ Health.

[b3-ehp-118-763] Babar IA, Slack FJ, Weidhaas JB (2008). miRNA modulation of the cellular stress response. Future Oncol.

[b4-ehp-118-763] Baccarelli A, Bollati V (2009). Epigenetics and environmental chemicals. Curr Opin Pediatr.

[b5-ehp-118-763] Baccarelli A, Martinelli I, Zanobetti A, Grillo P, Hou LF, Bertazzi PA (2008). Exposure to particulate air pollution and risk of deep vein thrombosis. Arch Intern Med.

[b6-ehp-118-763] Baccarelli A, Zanobetti A, Martinelli I, Grillo P, Hou L, Giacomini S (2007). Effects of exposure to air pollution on blood coagulation. J Thromb Haemost.

[b7-ehp-118-763] Bateson TF, Schwartz J (2004). Who is sensitive to the effects of particulate air pollution on mortality? A case-crossover analysis of effect modifiers. Epidemiology.

[b8-ehp-118-763] Bentwich I (2005). Prediction and validation of microRNAs and their targets. FEBS Lett.

[b9-ehp-118-763] Brauner EV, Forchhammer L, Moller P, Simonsen J, Glasius M, Wahlin P (2007). Exposure to ultrafine particles from ambient air and oxidative stress-induced DNA damage. Environ Health Perspect.

[b10-ehp-118-763] Brook RD, Franklin B, Cascio W, Hong Y, Howard G, Lipsett M (2004). Air pollution and cardiovascular disease: a statement for healthcare professionals from the Expert Panel on Population and Prevention Science of the American Heart Association. Circulation.

[b11-ehp-118-763] Chahine T, Baccarelli A, Litonjua A, Wright RO, Suh H, Gold DR (2007). Particulate air pollution, oxidative stress genes, and heart rate variability in an elderly cohort. Environ Health Perspect.

[b12-ehp-118-763] Chang CC, Hwang JS, Chan CC, Wang PY, Hu TH, Cheng TJ (2005). Effects of concentrated ambient particles on heart rate variability in spontaneously hypertensive rats. J Occup Health.

[b13-ehp-118-763] Chen CZ (2005). MicroRNAs as oncogenes and tumor suppressors. N Engl J Med.

[b14-ehp-118-763] Cheng Y, Liu X, Zhang S, Lin Y, Yang J, Zhang C (2009). MicroRNA-21 protects against the H(2)O(2)-induced injury on cardiac myocytes via its target gene PDCD4. J Mol Cell Cardiol.

[b15-ehp-118-763] Ciocco A, Thompson DJ (1961). A follow-up of Donora ten years after: methodology and findings. Am J Public Health Nations Health.

[b16-ehp-118-763] Corey LM, Baker C, Luchtel DL (2006). Heart-rate variability in the apolipoprotein E knockout transgenic mouse following exposure to Seattle particulate matter. J Toxicol Environ Health A.

[b17-ehp-118-763] Dai R, Phillips RA, Zhang Y, Khan D, Crasta O, Ahmed SA (2008). Suppression of LPS-induced Interferon-gamma and nitric oxide in splenic lymphocytes by select estrogen*-*regulated microRNAs: a novel mechanism of immune modulation. Blood.

[b18-ehp-118-763] Davis BN, Hilyard AC, Lagna G, Hata A (2008). SMAD proteins control DROSHA-mediated microRNA maturation. Nature.

[b19-ehp-118-763] De Palma G, Goldoni M, Catalani S, Carbognani P, Poli D, Mozzoni P (2008). Metallic elements in pulmonary biopsies from lung cancer and control subjects. Acta Biomed.

[b20-ehp-118-763] Descotes J (1992). Immunotoxicology of cadmium. IARC Sci Publ.

[b21-ehp-118-763] Frisch M, Klocke B, Haltmeier M, Frech K (2009). LitInspector: literature and signal transduction pathway mining in PubMed abstracts. Nucleic Acids Res.

[b22-ehp-118-763] Gurgueira SA, Lawrence J, Coull B, Murthy GG, González-Flecha B (2002). Rapid increases in the steady-state concentration of reactive oxygen species in the lungs and heart after particulate air pollution inhalation. Environ Health Perspect.

[b23-ehp-118-763] He L, Hannon GJ (2004). MicroRNAs: small RNAs with a big role in gene regulation. Nat Rev Genet.

[b24-ehp-118-763] Hurst DR, Edmonds MD, Scott GK, Benz CC, Vaidya KS, Welch DR (2009). Breast cancer metastasis suppressor 1 up-regulates miR-146, which suppresses breast cancer metastasis. Cancer Res.

[b25-ehp-118-763] Jardim M, Fry R, Jaspers I, Dailey L, Diaz-Sanchez D (2009). Disruption of microRNA expression in human airway cells by diesel exhaust particles is linked to tumorigenesis-associated pathways. Environ Health Perspect.

[b26-ehp-118-763] Kuo HW, Chang CL, Liang WM, Chung BC (1999). Respiratory abnormalities among male foundry workers in central Taiwan. Occup Med (Lond).

[b27-ehp-118-763] Labbaye C, Spinello I, Quaranta MT, Pelosi E, Pasquini L, Petrucci E (2008). A three-step pathway comprising PLZF/miR-146a/CXCR4 controls megakaryopoiesis. Nat Cell Biol.

[b28-ehp-118-763] Lewis BP, Burge CB, Bartel DP (2005). Conserved seed pairing, often flanked by adenosines, indicates that thousands of human genes are microRNA targets. Cell.

[b29-ehp-118-763] Li N, Xia T, Nel AE (2008). The role of oxidative stress in ambient particulate matter-induced lung diseases and its implications in the toxicity of engineered nanoparticles. Free Radic Biol Med.

[b30-ehp-118-763] Li Z, Hyseni X, Carter JD, Soukup JM, Dailey LA, Huang YC (2006). Pollutant particles enhanced H2O2 production from NAD(P)H oxidase and mitochondria in human pulmonary artery endothelial cells. Am J Physiol Cell Physiol.

[b31-ehp-118-763] Lin CS, Wang LS, Tsai CM, Wei YH (2008). Low copy number and low oxidative damage of mitochondrial DNA are associated with tumor progression in lung cancer tissues after neoadjuvant chemotherapy. Interact Cardiovasc Thorac Surg.

[b32-ehp-118-763] Liu X, Nelson A, Wang X, Kanaji N, Kim M, Sato T (2009). MicroRNA-146a modulates human bronchial epithelial cell survival in response to the cytokine-induced apoptosis. Biochem Biophys Res Commun.

[b33-ehp-118-763] Livak KJ, Schmittgen TD (2001). Analysis of relative gene expression data using real-time quantitative PCR and the 2^−ΔΔC_T_^ method. Methods.

[b34-ehp-118-763] Lu TX, Munitz A, Rothenberg ME (2009). MicroRNA-21 is up*-*regulated in allergic airway inflammation and regulates IL-12p35 expression. J Immunol.

[b35-ehp-118-763] MacNee W, Donaldson K (2000). How can ultrafine particles be responsible for increased mortality?. Monaldi Arch Chest Dis.

[b36-ehp-118-763] Magari SR, Schwartz J, Williams PL, Hauser R, Smith TJ, Christiani DC (2002). The association of particulate air metal concentrations with heart rate variability. Environ Health Perspect.

[b37-ehp-118-763] Meng F, Henson R, Lang M, Wehbe H, wari S, Mendell JT (2006). Involvement of human micro-RNA in growth and response to chemotherapy in human cholangiocarcinoma cell lines. Gastroenterology.

[b38-ehp-118-763] Meng F, Henson R, Wehbe-Janek H, Ghoshal K, Jacob ST, Patel T (2007). MicroRNA-21 regulates expression of the PTEN tumor suppressor gene in human hepatocellular cancer. Gastroenterology.

[b39-ehp-118-763] Messner B, Knoflach M, Seubert A, Ritsch A, Pfaller K, Henderson B (2009). Cadmium is a novel and independent risk factor for early atherosclerosis mechanisms and in vivo relevance. Arterioscler Thromb Vasc Biol.

[b40-ehp-118-763] Mishra KP (2009). Lead exposure and its impact on immune system: a review. Toxicol In Vitro.

[b41-ehp-118-763] Moschos SA, Williams AE, Perry MM, Birrell MA, Belvisi MG, Lindsay MA (2007). Expression profiling *in vivo* demonstrates rapid changes in lung microRNA levels following lipopolysaccharide-induced inflammation but not in the anti-inflammatory action of glucocorticoids. BMC Genomics.

[b42-ehp-118-763] Nel A, Xia T, Madler L, Li N (2006). Toxic potential of materials at the nanolevel. Science.

[b43-ehp-118-763] Papagiannakopoulos T, Shapiro A, Kosik KS (2008). MicroRNA-21 targets a network of key tumor-suppressive pathways in glioblastoma cells. Cancer Res.

[b44-ehp-118-763] Park SK, O-Neill MS, Vokonas PS, Sparrow D, Wright RO, Coull B (2008). Air pollution and heart rate variability: effect modification by chronic lead exposure. Epidemiology.

[b45-ehp-118-763] Pearce N, Checkoway H, Kriebel D (2007). Bias in occupational epidemiology studies. Occup Environ Med.

[b46-ehp-118-763] Perry MM, Moschos SA, Williams AE, Shepherd NJ, Larner-Svensson HM, Lindsay MA (2008). Rapid changes in microRNA-146a expression negatively regulate the IL-1beta-induced inflammatory response in human lung alveolar epithelial cells. J Immunol.

[b47-ehp-118-763] Roy S, Khanna S, Hussain SR, Biswas S, Azad A, Rink C (2009). MicroRNA expression in response to murine myocardial infarction: miR-21 regulates fibroblast metalloprotease-2 via phosphatase and tensin homologue. Cardiovasc Res.

[b48-ehp-118-763] Schmelzer C, Kitano M, Rimbach G, Niklowitz P, Menke T, Hosoe K (2009). Effects of ubiquinol-10 on microRNA-146a expression in vitro and in vivo. Mediators Inflamm.

[b49-ehp-118-763] Sen CK, Gordillo GM, Khanna S, Roy S (2009). Micromanaging vascular biology: tiny microRNAs play big band. J Vasc Res.

[b50-ehp-118-763] Shoenfelt J, Mitkus RJ, Zeisler R, Spatz RO, Powell J, Fenton MJ (2009). Involvement of TLR2 and TLR4 in inflammatory immune responses induced by fine and coarse ambient air particulate matter. J Leukoc Biol.

[b51-ehp-118-763] Suarez Y, Fernandez-Hernando C, Pober JS, Sessa WC (2007). Dicer dependent microRNAs regulate gene expression and functions in human endothelial cells. Circ Res.

[b52-ehp-118-763] Tarantini L, Bonzini M, Apostoli P, Pegoraro V, Bollati V, Marinelli B (2009). Effects of particulate matter on genomic DNA methylation content and iNOS promoter methylation. Environ Health Perspect.

[b53-ehp-118-763] Terasawa K, Ichimura A, Sato F, Shimizu K, Tsujimoto G (2009). Sustained activation of ERK1/2 by NGF induces microRNA-221 and 222 in PC12 cells. FEBS J.

[b54-ehp-118-763] Thum T, Gross C, Fiedler J, Fischer T, Kissler S, Bussen M (2008). MicroRNA-21 contributes to myocardial disease by stimulating MAP kinase signalling in fibroblasts. Nature.

[b55-ehp-118-763] Wang Z, Neuburg D, Li C, Su L, Kim JY, Chen JC (2005). Global gene expression profiling in whole-blood samples from individuals exposed to metal fumes. Environ Health Perspect.

[b56-ehp-118-763] Williams AE, Perry MM, Moschos SA, Larner-Svensson HM, Lindsay MA (2008). Role of miRNA-146a in the regulation of the innate immune response and cancer. Biochem Soc Trans.

[b57-ehp-118-763] Xiao C, Rajewsky K (2009). MicroRNA control in the immune system: basic principles. Cell.

[b58-ehp-118-763] Zeiher AM (1996). Endothelial vasodilator dysfunction: pathogenetic link to myocardial ischaemia or epiphenomenon?. Lancet.

